# The Buffalo Ophthalmological Club

**Published:** 1912-03

**Authors:** 


### The Buffalo Ophthalmological Club

Celebrated the 10th anniversary of its organization with a banquet at the Saturn Club, Saturday evening, February 17th.

The Ophthalmological Club was formed ten years ago with thirteen charter members, all from Buffalo. Since that time nine new members have been added from this city and seven from neighboring cities in western New York. Three members have died: Dr. Frank H. Coyle of Hornell and Drs. Julius Pohlman and Alvin A. Hubbell of Buffalo, leaving a present membership of twenty-six. The Club meets once a month with a very high percentage of attendance. Cases are presented, papers read and discussed and several prominent ophthalmologists from other cities have presented papers at various times.

Nearly the full membership was present at the banquet, at which Dr. Lucien Howe presided as toastmaster and the following formal toasts were responded to: The Dark Era. Pre-Ophthalmologist Club Days, by Dr. B. H. Grove; The Dawn of Buffalo Ophthalmologist Light, by Dr. Elmer Starr; Foreign Relations, by Dr. George Blackham of Dunkirk; Ideals for the Future, by Dr. F. Park Lewis; The Vacant Chairs, by Dr. Arthur G Bennett; and Reminiscences by Dr. Henry Y. Grant. All the members present participated in the festivities of the occasion.

